# Integrating Mechanisms in Thrombotic Peripheral Arterial Disease

**DOI:** 10.3390/ph15111428

**Published:** 2022-11-18

**Authors:** Magdolna Nagy, Paola E. J. van der Meijden, Julia Glunz, Leon Schurgers, Esther Lutgens, Hugo ten Cate, Stefan Heitmeier, Henri M. H. Spronk

**Affiliations:** 1 Department of Biochemistry, Cardiovascular Research Institute Maastricht, Maastricht University, 6229 ER Maastricht, The Netherlands; 2 Thrombosis Expertise Center, Heart and Vascular Center, Maastricht University Medical Center+, 6229 HX Maastricht, The Netherlands; 3 Cardiovascular Research, Bayer AG, 42117 Wuppertal, Germany; 4 German Centre for Cardiovascular Research (DZHK), Partner Site Munich Heart Alliance, 10785 Munich, Germany; 5 Institute for Cardiovascular Prevention (IPEK), Ludwig Maximilian’s University, 80539 Munich, Germany; 6 Experimental Cardiovascular Immunology Laboratory, Department of Cardiovascular Medicine, Mayo Clinic, Rochester, MN 55902, USA; 7 Department of Internal Medicine, Maastricht University Medical Center+, 6229 HX Maastricht, The Netherlands; 8 Center for Thrombosis and Hemostasis, Gutenberg University Mainz, 55122 Mainz, Germany

**Keywords:** peripheral artery disease, coagulation, thrombosis, atherosclerosis

## Abstract

Peripheral arterial disease (PAD), a manifestation of systemic atherosclerosis, is underdiagnosed in the general population. Despite the extensive research performed to unravel its pathophysiology, inadequate knowledge exists, thus preventing the development of new treatments. This review aims to highlight the essential elements of atherosclerosis contributing to the pathophysiology of PAD. Furthermore, emphasis will be placed on the role of thrombo-inflammation, with particular focus on platelet and coagulation activation as well as cell–cell interactions. Additional insight will be then discussed to reveal the contribution of hypercoagulability to the development of vascular diseases such as PAD. Lastly, the current antithrombotic treatments will be discussed, and light will be shed on promising new targets aiming to aid the development of new treatments.

## 1. Introduction

Atherosclerosis is a chronic, progressive thrombo-inflammatory vascular disease that occurs in all people, starting at a relatively young age, and disease progression depends on genetic background and a multitude of acquired risk factors. In Western society, most people, males and females alike, will not experience symptomatic atherosclerotic disease before the age of 65 [1], with increasing incidence of manifestations in the next few decades. In most subjects with atherosclerosis, symptomatic disease will occur based on critical atherosclerotic lesions within one vascular bed, e.g., myocardial ischemia due to thrombosis superimposed in atherosclerotic lesions in one or more coronary arteries. Other persons may experience symptoms related to cerebral ischemia caused by atherothrombotic occlusion in the carotid arteries or more upstream, resulting in transient ischemic attack (TIA) or ischemic stroke (excluding here thromboembolic strokes related to atrial fibrillation). Depending on the definition, atherosclerotic disease in the heart is regarded as central vascular disease, while atherosclerosis in other vascular beds is referred to as peripheral arterial disease (PAD). Another approach is to consider PAD primarily as a vascular disease related to the lower limbs, where the symptomatic presentation may be claudication or pain upon exercise, diminishing at rest. In general, however, one should consider that any presentation of atherosclerotic disease is indicative of the presence of significant atherosclerotic disease in other vascular beds as well, with the exception being atherosclerosis confined mostly to a single vascular bed.

Exact figures on the prevalence of PAD cannot be provided as the overall prevalence of PAD ranges between 1.8% and 25% depending on the population studied and the cut-off value of the Ankle Brachial Index (ABI) [2]. The prevalence increases with age from 3–10% amongst those aged 40–70 years to 10–20% for those aged 70 years and older [3]. Estimates of PAD incidence are rare; however, the ARIC Cohort Study reported a 10–13% age-standardized overall annual prevalence of PAD, accompanied with an age-standardized overall annual incidence of >25% per 1000 person-years [4]. In PAD of the legs, clinical severity is estimated with the Fontaine classification (Table 1), which is the primary determinant for referral to the vascular specialist and a starting point for clinical management.

Importantly, PAD of the legs is, even in the absence of symptoms when discovered by “chance”, associated with a high risk of cardiovascular morbidity (stroke, myocardial infarction, major adverse limb events (MALEs)) and mortality. For this reason, PAD diagnosis should always trigger substantial efforts to modify lifestyle (e.g., stop smoking and recommend exercise), reduce risk factors such as hypertension and hypercholesterolemia or diabetes, and institute antithrombotic medication to reduce the risk of cardiovascular complications. Interestingly, despite the increased risk of cardiovascular events in patients with PAD, there is marked heterogeneity among patients, and estimating the individual risk of vascular complications or life expectancy remains challenging. Various biomarkers have been tested in order to improve the selection of patients with the highest or lowest risk of vascular complications. In a systematic review, the fibrinolysis biomarker d-dimer was shown to predict cardiovascular mortality in patients with PAD [5,6]. However, because of a certain overlap between individuals, these single biomarker measurements are not diagnostically conclusive for individual risk classification. Furthermore, recent clinical studies have provided new and improved strategies for antithrombotic management; therefore, in this paper, we review the scientific knowledge on thrombo-inflammatory mechanisms in atherothrombosis, with a focus on biomarkers and antithrombotic management.

## 2. Atherosclerosis

### 2.1. Mechanisms

**Figure 1:**
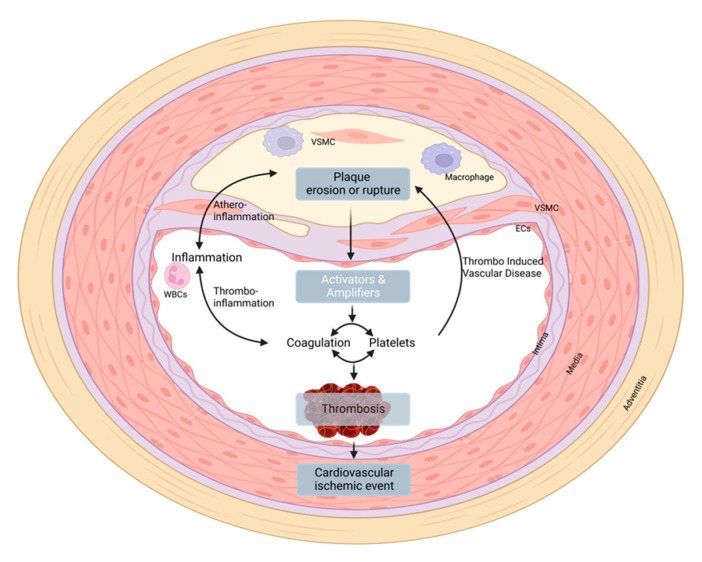
Schematic representation of interactions between atherosclerosis, inflammation, and thrombosis in peripheral arterial disease. Inflammation initiates or stimulates the development and progression of atherosclerosis, while the atherogenic process triggers inflammation (the athero-inflammation axis), which acts as key modulator of thrombosis (the thrombo-inflammation axis). Plaque and vascular components trigger the activation of platelets and the coagulation system, leading to thrombus formation. Activated platelets and ongoing coagulation further enhance inflammation, thereby indirectly influencing atherosclerosis. The direct effects of active coagulation factors and activated platelets on atherosclerosis are depicted in the “thrombo-induced vascular disease axis”, in which coagulation proteases directly affect atherogenic cells such as macrophages, smooth muscle cells, and endothelial cells. Created with Biorender.com accessed on 10 October 2022.

Although it is generally accepted that atherosclerosis is characterized by lipid accumulation and inflammation in the intimal layer of the arterial vessel wall, its pathogenesis has remained controversial for decades. Atherosclerosis is complex and is believed to be caused by a concert of interactions between lipids, immune cells, platelets, and the endothelium, resulting in atherosclerotic plaque development and pro-inflammatory signaling in the arterial wall (Figure 1). The release of chemokines, cytokines, and extracellular vesicles (EVs) further drives atherosclerotic plaque growth by continuous recruitment of immune cells. When atherosclerotic lesions grow, hypoxia results in cell death, necrotic core formation, and plaque angiogenesis. Moreover, these interactions result in the phenotypic switching of vascular smooth muscle cells (VSMCs), causing migration, proliferation, and subsequent extracellular vesicle-initiated calcification [7]. Besides inflammation and lipids, activated coagulation factors (pre-thrombotic state) have now been accepted as key factors in the genesis and progression of vascular disease [8]. Activated coagulation factors (F) IIa and Xa signal through a family of cellular protease-activated receptors (PARs), PAR1–4. PARs are widely distributed on vascular cells under normal conditions and are overexpressed during atherogenesis [9]. Proteolytic activation of PARs by either FIIa or FXa results in the activation of a canonical G-protein pathway, and downstream signaling pathways influence multiple transcription-regulated, cell-specific events including proliferation, inflammation, migration, adhesion, and apoptosis [8].

### 2.2. Vascular Bed Specificity

The mechanisms for atherosclerotic plaque development are grossly similar along the entire arterial tree; however, plaques in the different arterial beds have different features. These differences depend on local factors specific to the site, including anatomy, hemodynamics, and embryonic origin. For example, carotid and coronary atherosclerosis overlap in many key areas but also differ in many other aspects, including endothelial cell heterogeneity [10]. Systemic factors, such as blood lipid levels, are similar in coronary and carotid atherosclerosis for obvious reasons. The hemodynamic environment, however, is very different in the two vascular beds. The amount of flow through the carotid artery is mostly determined during the systolic phase. In contrast, flow through coronary arteries is only present during the diastolic phase, as the smaller coronary vessels are closed by the ventricular pressure during the systolic phase. Moreover, the embryonic origin is different as coronary arteries are derived from the neural crest whereas the carotid arteries are derived from the third pharyngeal arch. Compared to carotid atherosclerotic plaques, coronary plaques are more affected by atherosclerosis, most likely due to the greater vasa vasorum and risk for intraplaque hemorrhage [11]. After plaque rupture, the mean fibrous cap thickness is much thicker in ruptured carotid plaques (~200 µm) as compared to ruptured coronary plaques (~65 µm), most likely due to the stronger pull, and thus earlier rupture, on the fibrous cap caused by different hemodynamics in the carotid artery [12,13]. Interestingly, VSMCs show high heterogeneity across and within vascular areas, which implicates specific functions in response to injury and atherosclerosis [14].

### 2.3. Rupture vs. Erosion

The progression of plaque and plaque build-up is referred to as thickening of the vessel wall, ultimately narrowing the lumen of the vessel. Most atherosclerotic plaques stay dormant, and atherosclerotic burden is clinically assessed as the amount of calcification by computed tomography [15]. Vascular calcification is not limited to the coronary arteries, and calcification of any vascular bed is associated with an increased risk of vascular morbidity or mortality [16]. Unstable plaque is still the major cause of acute clinical sequelae of atherosclerosis. However, these thin fibrous plaques often persist for many years without rupturing and causing a clinical event [17]. The genesis and rupturing of atherosclerotic plaques not only depend on local environmental factors but also on the interaction with the fluid phase of blood—for example, procoagulant microparticles [18] and activated coagulation factors [8].

As atherosclerotic disease progresses, plaques will cause ischemic syndromes via two main mechanisms. Plaque rupture involves the formation of a thrombus as a result of disruption of the fibrous cap overlying a large lipid rich necrotic core. This way, thrombosis contributes to plaque and vessel remodeling, and consequently to stenosis; in the acute phase, occlusion of the vessel may occur. Plaque erosion refers to the formation of a thrombus in an area of endothelial denudation adjacent to an atherosclerotic plaque without disruption of the fibrous cap overlying a (deep) small necrotic core. Thrombus formation resulting from the exposure of VSMCs and extracellular matrix to the blood can also cause occlusion of the artery. Over the years, pathologists have noted that the frequency of plaque erosion has increased. These changes over time are possibly due to the increased statin use and decreased tobacco use in recent years [17].

Whereas plaque rupture mostly occurs through active inflammation, followed by the release of proteolytic enzymes and degradation of the fibrous cap, the pathogenesis of plaque erosion is less clear. However, it is thought that in regions of disturbed flow, Toll-like receptor 2 (TLR2) is activated on endothelial cells. Ligation of TLR2 induces the production of IL-8, promoting neutrophil adhesion and recruitment, which causes endothelial cell damage, ultimately resulting in the exposure of the fibrous cap to the blood. This exposure activates platelets, causing them to release pro-inflammatory mediators (further aggravating the process leading to endothelial cell death) and blockers of fibrinolysis (increasing the durability of clots). As proposed by the groups of Libby and Pasterkamp, clots resulting from plaque erosion are platelet-rich ‘white’ clots, whereas clots resulting from plaque rupture can be a mix of white and erythrocyte/fibrin-rich ‘red’ clots [17].

## 3. Thrombo-Inflammation

### 3.1. Modulators of Platelet Function

**Figure 2:**
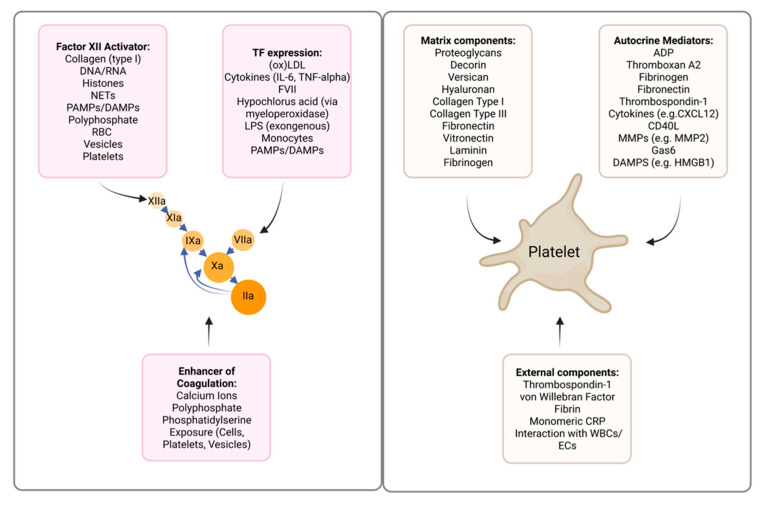
Atherosclerotic plaque-associated activators and mediators of coagulation and platelets. Created with Biorender.com accessed on 10 October 2022.

Atherosclerotic lesions contain multiple vascular and blood-derived components that mediate the adhesion and/or activation of platelets in the circulation (Figure 2). Here, we highlight several platelet-activating components related to atherosclerotic plaques. Depending on the plaque phenotype, being either a stable, ruptured, or eroded atherosclerotic lesion, specific proteoglycans of the extracellular matrix can be enriched or deprived [19]. The proteoglycan decorin, present in stable plaques, supports platelet adhesion via integrin α2β1, leading to tyrosine phosphorylation events that trigger inside-out activation of integrin αIIbβ3 and thereby enhance the binding of fibrinogen [20,21]. In lesions with plaque erosion, versican and the glycosaminoglycan hyaluronan are abundantly present [19]. It was demonstrated that vascular versican can promote the adhesion of flowing platelets, although the receptor(s) responsible for this interaction remains to be discovered [22]. One of the cellular receptors for hyaluronan, CD44, was demonstrated by immunohistochemical staining of eroded coronary plaques to be at the plaque–thrombus interface [23], where platelet-expressed CD44 can mediate adhesion to hyaluronan [24].

Thrombospondin-1 (TSP1) is a glycoprotein involved in various biological processes which, by interacting with vascular factors (e.g., vWF and fibrinogen), can modulate platelet activity. Platelets not only secrete TSP1 from their α-granules upon activation but also bind TSP1 through several receptors, such as the glycoprotein Ib-V-IX complex, CD47, integrins, and CD36 [25]. Platelet-secreted TSP1 was shown to enhance thrombus formation and stabilization in a CD36-dependent manner [26]. In addition to TSP1, CD36 acts as the main platelet receptor for oxidized low-density lipoproteins (oxLDLs) [27,28], which are abundantly present in plaque areas. CD36–oxLDL interaction enhances platelet activation and collagen-dependent thrombus formation under flow [28]. Next to directly promoting platelet activation, oxLDL induces CD36-dependent production of reactive oxygen species (ROS), which desensitizes the inhibitory action of cGMP signaling [29].

Activated platelets secrete secondary mediators such as ADP, CD40 ligand (CD40L), cytokines, and matrix metalloproteinases (MMPs), which, besides enhancing platelet activation, can modulate inflammation and atherosclerosis. CD40L is expressed on the platelet surface after stimulation, where it is cleaved by extracellular proteases such as MMPs, generating the soluble form (sCD40L) [30,31]. Soluble CD40L was reported to stabilize arterial thrombi in an experimental thrombosis model, independent of its widely expressed CD40 receptor, by acting as a ligand for integrin αIIbβ3, triggering outside-in signaling [32,33]. Another study proposed that sCD40L enhances platelet activation and aggregation entirely through CD40-related TRAF (TNF receptor-associated factor) signaling [34]. Although ex vivo thrombus growth on plaque material was impaired with both CD40L-deficient and CD40-deficient murine blood, CD40L was shown to act partly independently of CD40 [35].

Carotid atherosclerotic plaques were demonstrated to have a high content of active MMP-2, which plays a role in extracellular matrix degradation, neutrophil activation, and platelet activation [36]. Platelets also release MMP-2, and in in vivo arterial thrombosis models as well as collagen-dependent whole blood perfusion experiments, platelet-associated MMP-2 promoted thrombus formation by amplifying platelet activation [37,38]. Intracellular MMP-2 appears to facilitate platelet aggregation via hydrolysis of talin, which is involved in the activation of integrin αIIbβ3 [39]. Recently, active MMP-2 was shown to cleave the platelet thrombin receptor PAR1, leading to the priming of platelets for full activation by other stimuli as well as activating endothelial PAR1, leading to the expression of adhesion molecules [40,41].

Platelets are a major source of the chemokine CXCL12 (stromal cell-derived factor-1α, SDF-1α), which is released from α-granules following activation. CXCL12 binds to its Gαi-coupled receptor CXCR4 and potentiates platelet activation and collagen-induced thrombus formation, in part via synergistic effects of ADP and thromboxane A2 [42,43]. Interestingly, recent evidence shows that CXCL12 enhances platelet lipid uptake, such as LDL and oxLDL, via CXCR4 and CXCR7, favoring the intracellular generation of oxidized lipid metabolites that promote the prothrombotic functions of platelets [44].

### 3.2. Tissue Factor: Factor VII Pathway

Direct activators of coagulation factors, other than FXII and tissue factor (TF), are hardly known in atherosclerotic lesions, and if described in the literature, they have not been confirmed in recent decades. Therefore, the assumption is that coagulation is mainly activated through the extrinsic (TF:FVII) or intrinsic (FXII) pathway. The key players of the TF pathway are all expressed or localized within the atherosclerotic lesion. TF, FVII, and FX are mainly associated with macrophages, foam cells, and smooth muscle cells, whereas FX is also recognized on endothelial cells [45,46]. The mechanisms leading to in vivo activation of FVII are not known, although two main pathways have been suggested, including allosteric activation, and limited proteolytic activation by either FXa or FIXa [47,48,49]. Despite heterogeneity in association with various cells, the presence of almost all coagulation factors within the atherosclerotic lesions suggests that localized cellular coagulation compartments are contributing to fibrin formation and/or activation of cellular PARs [46].

### 3.3. Activators of the Factor XII-Dependent Intrinsic Coagulation Pathway

Several physiological activators of FXII that promote coagulation have been identified inside the plaque area or in close vicinity of the forming thrombus. Collagen type I not only acts as a potent platelet activator but also binds and activates FXII [50]. In addition, laminin present in the basement membrane accelerates fibrin formation in an FXII-dependent manner [51]. In an in vivo arterial thrombosis model induced by plaque rupture, FXIIa was important for stabilization of the luminal regions of platelet-fibrin thrombi [52]. Activated platelets present in thrombi can induce activation of FXII via the release of polyphosphates (polyPs) from their dense granules [53]. Recent evidence indicates that not soluble polyP but rather polyP bound to Ca^2+^ in nanoparticles on the platelet surface is responsible for FXII activation [54]. Furthermore, extracellular vesicles derived from platelets as well as red blood cells can initiate FXII-dependent thrombin generation through direct activation of FXII and prekallikrein [55,56]. Additionally, extracellular histones [57,58] and DNA/RNA have been shown to activate FXII [59], which could be in part mediated by neutrophil extracellular traps (NETs) [60,61], which are primarily composed of DNA and histones. Cholesterol crystals within atherosclerotic lesions appear to induce suicidal NETosis through sterile inflammation [62]. Despite there being more evidence present supporting NET-induced FXII activation, there is some evidence showing the expression of functional TF on NETs in myocardial infarct [63].

### 3.4. Cell–Cell Interactions

**Figure 3:**
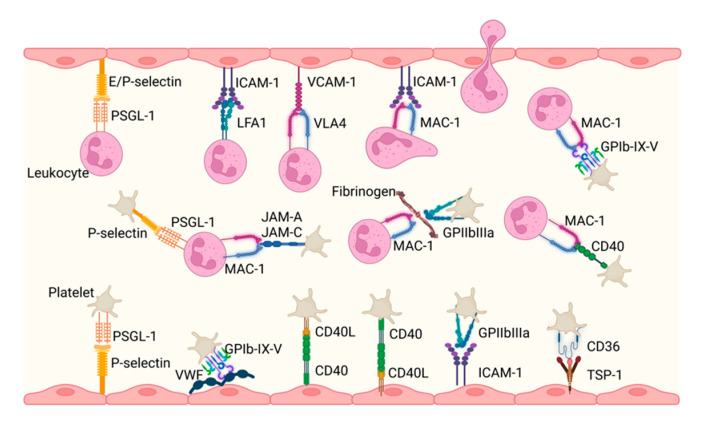
Schematic representation of interactions between platelets, leukocytes (e.g., neutrophils), and endothelial cells in thrombo-inflammation. Platelets interact with both endothelial cells and leukocytes through a variety of receptors/adhesion molecules. Besides activation of platelets, the interactions may induce endothelial cell responses, including apoptosis and inflammation. The interactions between platelets and leukocytes might result in prothrombotic platelet/leukocyte aggregates as well as activation of coagulation through released components of neutrophils. Created with Biorender.com accessed on 10 October 2022.

The main receptors involved in the interplay between platelets and endothelial cells, leukocytes and endothelial cells, and platelets and leukocytes are well known and extensively reviewed elsewhere and have been schematically presented in Figure 3 [64,65]. Examples of receptor–ligand interactions between platelets and endothelial cells include GP1b–vWF; P-selectin–PSGL-1; CD40–CD40L; and the binding of integrin αIIbβ3 to von Willebrand Factor, αvβ3, or ICAM-1. The importance of the P-selectin–PSGL-1 axis for cell–cell binding is recognized by the fact that it mediates the initial interaction between platelets and leukocytes. This interaction is further established through binding of the leukocyte αMβ2/MAC1 to the platelet receptors GP1b, JAM-A, JAM-C, αIIbβ3, or CD40L. Furthermore, leukocytes are captured on endothelial cells through binding of PSGL-1 to E- or P-selectin and via deposition of platelet-derived chemokines (reviewed in [66,67,68]). Adding to the complexity of cellular interactions involved in arterial thrombosis, leukocytes can bind platelets adhered to the endothelium [69]. Driven by chemokines, leukocytes transverse and migrate into the extracellular space of the arterial vessel wall. This migration process is mediated by PECAM-1, JAM-1, and VE-Cadherin. The contribution of leukocytes within the atherosclerotic vessel wall to thrombosis is not clear yet, although a role in plaque progression is evident [70].

Several recent studies have elucidated important mediators of these cell–cell interactions contributing to atherothrombosis. One novel mediator is protein disulfide isomerase (PDI), secreted by activated platelets and endothelial cells, thereby supporting the binding of the two cell types. Using recombinant PDI variants and in vivo thrombosis models, it was demonstrated that PDI reduces disulfide bonds on plasma vitronectin, resulting in enhanced thrombosis through binding of vitronectin to its integrin receptors on endothelium (αvβ3) and platelets (αIIbβ3) [71].

Activated platelets also have the ability to induce NET formation [72], although the underlying mechanism is unclear. One of the potential mechanisms depends on the delivery of high mobility group box 1 (HMGB1), a damage-associated molecular pattern, to the neutrophils via TLR4/9 [73] or Receptor for Advanced Glycation End products (RAGE) [74]. In addition, platelet-derived HMGB1 promotes thrombosis by enhancing platelet activation and aggregation in a TLR4/MyD88-dependent manner [75]. In turn, NETs, composed of decondensed chromatin and granular proteins, have been identified to promote thrombus formation through platelet-independent and -dependent mechanisms [61]. Not only do NETs provide a scaffold for the adhesion of platelets, but their components can also activate platelets as well as proenzymes of the coagulation cascade (reviewed in [76]). Histones H3 and H4 activate platelets either via a direct interaction with TLR2 and -4 [77], or indirectly via fibrinogen [78]. On the other hand, NETs induce contact activation of FXII [79] and alter the overall balance towards a procoagulant state by interfering with the anticoagulant factors protein C [57] and tissue factor pathway inhibitor (TFPI) [72].

Furthermore, red blood cells (RBCs) have been long disregarded in relation to cardiovascular diseases such as atherosclerosis, but emerging evidence demonstrates that RBCs are not only bystanders. RBCs play important immunomodulatory functions in atherosclerosis as a consequence of the interplay between cell–cell interactions, production of oxygen species, changes in oxidative state, and release of their hemoglobin. The role of RBCs has also been associated with plaque instability through their cholesterol content (reviewed in [80,81,82,83]).

Dann et al. performed platelet RNA sequence profiling in patients with symptomatic PAD and revealed a significant upregulation of myeloid-related protein (MRP)-14 mRNA with concomitant enrichment of MRP-14 protein. MRP-14 is suggested to enhance the expression of P-selectin on activated platelets, which promotes the interaction with monocytes. Interestingly, the level of circulating MRP-14 was associated with adverse cardiovascular and limb events [84]. Furthermore, Newman et al. performed whole blood RNA sequence profiling on patients with symptomatic PAD and controls and revealed blood modules enriched for coagulation and immune activation in PAD patients [85]. Another study provided evidence that hypercholesterolemia in mice increases circulating platelet–monocyte aggregates and plaque platelet–macrophage aggregates. Via this interaction, platelets promote a pro-inflammatory myeloid phenotype via Socs3 expression, which induces inflammatory cytokine production. In patients with PAD, the link between platelet activity and the SOCS3-mediated inflammatory phenotype was confirmed [86].

## 4. Thrombo-Induced Vascular Disease

The notion that a hypercoagulable state contributes to atherosclerosis stems from several key experimental studies applying atherosclerosis models in combination with altered coagulation (reviewed in [87]). Atherogenic animals with a hypercoagulable genotype, including FV Leiden mutation, protein C deficiency, thrombomodulin pro/pro mutation, or TFPI deficiency, demonstrated a clearly increased atherosclerotic burden. In line with this, a recent genome-wide association study (GWAS) on PAD genetics revealed significant associations for the FV Leiden mutation with PAD risk and severity, supporting the importance of coagulation activity for atherogenesis in the peripheral vascular bed [88]. A broader role of coagulation enzymes in cardiovascular disease is illustrated by an increased susceptibility to atrial fibrillation in animals with the hypercoagulable genotype induced by the thrombomodulin pro/pro mutation [89]. Despite these observations, evidence from clinical studies is not yet conclusive. Clearly, markers of active thrombin generation such as TF, thrombin–antithrombin complexes, prothrombin fragment 1.2, and activation peptides of FIX have been associated with cardiovascular disease. Furthermore, thrombophilia genotypes including FV Leiden mutation or deficiencies in antithrombin, protein C, or protein S are moderately associated with cardiovascular disease, especially in young patients aged <45 years [90,91]. In contrast, hemophilia (FVIII or IX deficiency) does not protect against atherosclerosis, while conflicting results were seen regarding protection for cardiovascular mortality [87]. Since patients with hemophilia will often receive factor replacement therapy, it remains difficult to unequivocally assess any protective effects.

A “vascular protective” action of anticoagulants stems from experimental observations that direct inhibition of coagulation enzymes limits the development of atherosclerosis in pre-clinical studies. Both direct thrombin and factor Xa inhibition reduced the development of atherosclerosis in ApoE*^-/-^* mice [92,93,94,95,96]. Own data showed even plaque regression in atherosclerotic mice treated with rivaroxaban [97]. In summary, both clinical and pre-clinical studies suggest that inhibition of coagulation has a “vascular protective” effect, thereby demonstrating a direct link between coagulation enzymes and atherogenic processes.

The mechanisms by which mainly coagulation enzymes FXa and thrombin contribute to cardiovascular disease are not fully understood yet. As mentioned before, thrombin and FXa can activate PARs. Today, four subtypes are recognized: PAR1–4 [98], of which PAR1 and PAR2 are expressed in atherosclerotic lesions [99]. Thrombin, FVIIa, FXa, and the anticoagulant activated protein C (APC) induce cellular processes through activation of PARs. Thrombin and APC mainly activate PAR1, whereas FXa can activate both PAR1 and PAR2 [100]. Thrombin can induce both pro- and anti-inflammatory signals in the same cell, mainly driven but not limited by concentration, duration of activation, and localization. Adding to the complexity is PAR1-dependent biased signaling in which APC acts in a cytoprotective manner and thrombin in a cytotoxic direction [101].

PAR signaling may induce a phenotypic switch of endothelial cells (ECs), VSMCs, and macrophages directly, thereby contributing to cardiovascular disease. Whereas deletion of PAR1 on an atherosclerotic background did not alter the phenotype dramatically, absence of PAR2 proved to be cardiovascular protective on both ApoE^-/-^ and Ldlr^-/-^ backgrounds as demonstrated by attenuated atherosclerosis [102,103,104]. Whether this suggests that FXa rather than thrombin is involved is not clear yet. Other candidate agonists such as MMPs may also be involved. Limited clinical evidence suggests that DOAC treatment may indeed provide a degree of plaque stabilization as compared to either no anticoagulants or VKA [105].

Overall, the contribution of variation in coagulation activity to atherosclerosis may become clinically meaningful when fine-tuned antithrombotic therapies not only reduce atherothrombotic risk but also reduce atherosclerosis progression or even induce regression of existing atherosclerosis. The challenge is to unravel the complex interplay between coagulation enzymes and their receptors as well as to better understand the potentially opposing effects on atherogenesis.

## 5. Treatment Options

### 5.1. Current Antithrombotic Therapy in PAD

In patients with symptomatic atherosclerosis, anti-platelet agents have been the mainstay of (secondary) prevention. In patients with symptomatic PAD (of the lower extremities), an anti-platelet drug is prescribed lifelong, with a slight preference for clopidogrel based on the CAPRIE trial, which showed some advantage of clopidogrel over aspirin in the PAD subgroup of the trial [106]. In case of clinical suspicion of clopidogrel “insensitivity” (also labeled “resistance”), ticagrelor could be an alternative option given its different receptor binding site [107] and potential beneficial therapeutic application [108].

Following a percutaneous intervention, dual anti-platelet platelet inhibition (DAPT) can be prescribed, typically for no longer than 1 month due to increased bleeding risk, followed by single APT lifelong. Revascularization with synthetic graft material usually does not change APT prevention. Oral anticoagulation (VKA) is only indicated in patients with PAD that require venous bypass grafting, for a limited time (followed by APT). For patients with carotid artery atherosclerosis as a manifestation of PAD, APT with clopidogrel is routinely prescribed indefinitely, with aspirin (±dipyridamole) as an alternative option. Only following stent placement for symptomatic carotid artery disease, a 4-week period of DAPT is recommended [109]. In general, there are no other reasons to prolong DAPT in patients because of the increased major bleeding observed [110].

Until recently, oral anticoagulation was recommended for none of the manifestations of systemic arterial vascular disease unless a second indication such as AF existed. This has changed as recent clinical trials suggest that antithrombotic agents, besides reducing thrombosis risk, are “vascular protective”, thereby providing clinical support for the above-mentioned experimental observations. These potential atheroprotective effects of direct oral anticoagulants with or without anti-platelet therapy stem from observations in the phase III ATLAS study, which demonstrated that rivaroxaban treatment reduced death from cardiovascular disease, myocardial infarction, and stroke [111]. Data from the phase III COMPASS trial indicate an improved cardiovascular survival in patients with chronic coronary syndrome and/or PAD treated with rivaroxaban 2.5 mg plus aspirin as compared to aspirin only. Overall, patients with stable CAD or PAD receiving a combination of rivaroxaban and aspirin had a combined relative risk reduction in major cardiovascular events of almost 25% compared to subjects receiving aspirin alone [112]. This regimen was included in the 2019 ESC guidelines for diagnosis and management of chronic coronary syndromes.

The Efficacy and Safety of Rivaroxaban in Reducing the Risk of Major Thrombotic Vascular Events in Subjects with Symptomatic Peripheral Artery Disease Undergoing Peripheral Revascularization Procedures of the Lower Extremities (VOYAGER PAD [113]) trial confirmed the clinical importance of the dual pathway inhibition (DPI) approach, not only in the chronic phase but also in the acute stage after revascularization as well as in the prevention of venous thrombotic events [113,114]. The marked efficacy of DPI illustrates the importance of both the platelet and coagulation pathways in atherothrombosis and possibly also in the process of atherosclerosis, underlying plaque instability. Interestingly, in a recent meta-analysis, DPI consisting of aspirin and a low dose of rivaroxaban appeared to be superior to APT and DAPT in preventing major cardiovascular and adverse limb events in patients undergoing peripheral vascular intervention, and this could be considered for long-term prevention [115]. Nevertheless, several practical questions remain, including reasons for switching from single APT with clopidogrel to DPI with rivaroxaban and aspirin, as these two compounds were not directly compared in clinical trials.

### 5.2. New Targets

**Figure 4:**
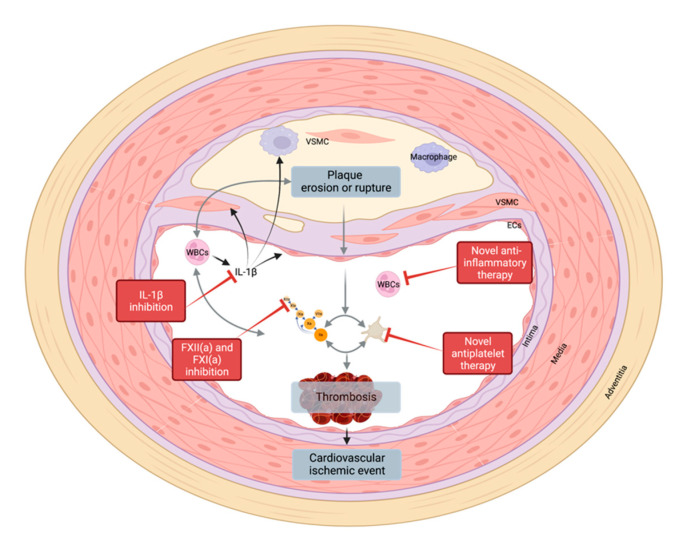
Indication of the main therapeutic targets providing possible approaches to combat atherosclerosis. Created with Biorender.com accessed on 10 October 2022.

As the use of current antithrombotics is often complicated by the associated increase in bleeding risk, therapies are therefore aimed at finding the ‘sweet spot’ between efficacy and safety. One focus in the development of next-generation antithrombotic drugs is the identification of safer approaches that target thrombosis without affecting hemostasis. Inhibition of FXI(a) or FXII(a) may offer such profiles. Future therapies may extend beyond the prevention of thrombosis-induced vascular occlusion as a final event by tackling the initiation of atherothrombosis and its link to concomitant pathological processes such as vessel calcification (Figure 4). This will bring the interactions between the diseased vessel wall and the different components in the blood to the forefront. Thus far, it has been challenging to discover good targets due the complexity, potential redundancies, and often missing tools. However, this may hold the option for more specific therapies or even patient-tailored approaches.

As stated above, inflammatory processes play an important role in vascular diseases. Drugs inhibiting prominent inflammatory pathways such as the anti-interleukin (IL)-1β antibody canakinumab improved the pain-free walking distance in PAD patients within 3 months compared to a placebo but did not influence plaque progression [116]. Further properly powered studies are needed to confirm these positive effects. Besides systemic inflammatory pathways, cytokines that are locally increased in the atherosclerotic lesion may be of special interest. ST2 from the IL-33-ST2 axis seems to be involved in the plaque-related upregulation of TF expression and activity on monocytes [117] and thereby contributes to a prothrombotic state, which explains the use of soluble ST2 as a potential biomarker. However, as with many other cytokines which have a spectrum of interaction partners and functions, IL-33 also has cardioprotective properties, and total inhibition might be harmful and not suitable for chronic treatment [118].

In the class of proteolytic enzymes—important in potentially pathological tissue remodeling—some of these enzymes, such as Pappalysin-1 (PAPP-A1), are also highly expressed in eroded and ruptured plaques and associated with ACS [119]. Further in vitro studies linked PAPP-A1 directly to increased expression of tissue factor on endothelial cells [120]. As for the cytokines, different functions are also described for PAPP-A1 in this case a plaque-stabilizing role, which complicates its complete inhibition for atherothrombosis prevention.

These few examples out of the tremendous number of involved pathways, which still need to be unraveled, demonstrate some of the challenges on our way to identifying the next generation of PAD therapy.

## 6. Conclusions

Despite the attention PAD has received on the cellular level, there are still many unanswered questions. More in vivo and in vitro studies are needed to provide in-depth knowledge on the potential impact of immunomodulation and other novel treatment options on plaque progression in PAD. From the clinical and translational points of view, one of the major challenges in understanding the outcomes of the current studies lies in the lack of coherent datasets and study populations. Currently, no PAD-specific biomarkers have been identified, which in part may be due to the complexity of the disease, with many underlying conditions hindering the biomarker profiles. Therefore, the focus should be not on finding one biomarker but rather on finding a biomarker profile that describes the PAD phenotype of the patient the most.

## Figures and Tables

**Table 1:** Fontaine classification.

Grade	Symptoms
Stage I	Asymptomatic, incomplete blood vessel obstruction
Stage II	Mild claudication pain in limb
Stage IIA	Claudication at a distance > 200 m
Stage IIB	Claudication at a distance < 200 m
Stage III	Rest pain, mostly in the feet
Stage IV	Necrosis and/or gangrene of the limb

## Data Availability

Data sharing not applicable.
